# Integration of Next Generation Sequencing Data to Inform Survival Prediction of Patients with Spine Metastasis

**DOI:** 10.3390/cancers17132218

**Published:** 2025-07-02

**Authors:** Alexandra Giantini-Larsen, Alexander D. Ramos, Axel Martin, Katherine S. Panageas, Caroline E. Kostrzewa, Zaki Abou-Mrad, Adam Schmitt, Jacqueline F. Bromberg, Anton Safonov, Charles M. Rudin, William Christopher Newman, Mark H. Bilsky, Ori Barzilai

**Affiliations:** 1 Department of Neurological Surgery, Memorial Sloan Kettering Cancer Center, New York, NY 10065, USA; 2 Department of Neurological Surgery, New York Presbyterian Hospital, Weill Cornell Medical Center, New York, NY 10065, USA; 3 Department of Epidemiology and Biostatistics, Memorial Sloan Kettering Cancer Center, New York, NY 10065, USA; 4 Department of Radiation Oncology, Memorial Sloan Kettering Cancer Center, New York, NY 10065, USA; 5 Department of Medicine, Memorial Sloan Kettering Cancer Center, New York, NY 10065, USA

**Keywords:** genomics, spine metastasis, machine learning

## Abstract

**Background/Objectives**: Spinal metastatic disease is a life-altering problem for individuals with cancer. Prognostication is key for tailored treatment of spinal metastases. This manuscript provides a comprehensive overview of the genomic profiles of metastatic spine tumors and investigates the potential of mutational data to stratify overall survival (OS) across various histologies. **Methods**: This is a cohort study of consecutive patients with spine metastatic disease whose tumors were sequenced on a next generation sequencing platform; a machine learning (ML) algorithm was used to stratify OS risk. **Results**: Targeted sequencing and stratification of OS risk of 282 spine metastases (breast (84), non-small cell lung (56), prostate (49), other (93)) was performed. *TP53* (HR 1.80; 95% CI 1.26, 2.56) and *KEAP1* (HR 3.95, 95% CI 2.24, 6.98) mutations were associated with poor survival across the entire cohort in univariate Cox proportional hazards models. The ML algorithm categorized breast cancer metastasis into low- and high-risk groups, revealing a median OS of 71 compared to 22 months (HR 3.3, *p* < 0.001). *TP53* mutations and *ESR1* mutations conferred poor prognosis. In lung cancer, low- and high-risk groups with median OS of 30 and 6 months (HR 8.3, *p* < 0.001), respectively, were identified with poor prognosis linked to *MET* amplification. No significant prognostic associations were identified for spinal prostate metastases. **Conclusions**: Metastatic spine tumor molecular data allows for the identification of prognostic groups. We present an open-source machine learning algorithm utilizing genomic mutational data that may aid in prognostication and tailored decision making.

## Simple Summary

Genomic mutational data is critical for prognostication in oncology. Spinal metastasis mutational data should be incorporated into the tailored decision-making algorithm for those with metastatic disease to the spine. Incorporation of spinal mutational data will improve stratification of patient prognoses, accuracy of prognostication, and aid in selection of multidisciplinary approaches to the treatment of metastatic spine disease. This study utilized large-scale sequencing data to represent the genomic landscape of spinal metastases to define prognostic groups for patients with metastatic disease to the spine from breast, non-small cell lung, and prostate cancer. To our knowledge, this is the first study dedicated to deciphering the genomic landscape of spinal metastasis and utilizing a machine learning algorithm for survival stratification based on high- and low-risk prognostic groups for the most common cancers metastasizing to the spine.

## 1. Introduction

An estimated 40% of cancer patients will experience metastasis to the spine over the course of their disease [1]. As the duration of survival for cancer patients improves due to highly active targeted therapies, effective management of spinal metastases is an increasingly critical clinical issue [2]. Management of spinal disease includes a combination of systemic therapy, radiation therapy, and in some cases, surgery [2,3,4]. The integration of multidisciplinary approaches has yielded an improvement in overall survival rates of patients affected by metastatic spinal disease [5]. However, accurately predicting patient prognoses in this population remains challenging, especially when considering whether surgical intervention is appropriate. Ensuring accurate prognostication is a key consideration in guiding appropriate management strategies for patients with metastatic disease to the spine. To this end, scoring systems and nomograms have been developed based on factors such as primary tumor type and systemic disease burden [6,7]. Nevertheless, none of these predictive tools incorporate tumor mutational data as part of the prognostication.

One of the most significant advances in clinical oncology over the past decade is the integration of comprehensive tumor genotyping into treatment algorithms and risk stratification for patients with a variety of solid tumors. Genotyping allows for identification of appropriate targeted therapies, such as EGFR and CDK4/6 inhibitors, which have dramatically influenced survival outcomes [8,9]. However, despite these advances, there exists limited knowledge about the genomic landscape of spinal metastases, and there are few reports of integration of genetic data aiding in prognosis. Recent large-scale efforts to better characterize human cancers and metastatic disease have included few tumor samples derived from bone or spine [10,11,12].

Here we report data derived from MSK-IMPACT (Memorial Sloan Kettering-Integrated Mutation Profiling of Actionable Cancer Targets) [13], a next generation sequencing platform of actionable cancer-related genes, to describe the genomic landscape of spinal metastases across multiple tumor types. This genomic data was then used to train a machine learning algorithm [14] to stratify patients into high- and low-risk prognostic groups for the most common cancers metastasizing to the spine.

## 2. Materials and Methods

### 2.1. Study Population

This is a retrospective analysis of prospectively collected, consecutive, tumor samples obtained from spinal metastases at a single institution from 2000–2019. Patients who had metastatic spine tumor samples that were obtained as part of routine clinical care and that were profiled by MSK-IMPACT were identified. CBioPortal was utilized [15,16,17]. This encompassed spinal samples acquired from bone biopsy and epidural tumors excised during surgical procedures; however, this analysis excluded intradural and intramedullary tumors. Tumors were required to have a purity of at least 30% to be included in our analysis. This study was approved by the institutional review board (IRB) of MSK, and informed consent for biospecimen collection and analysis was obtained from all patients. Data reviewed included patient demographics such as age, gender and tumor histology as well as procedural data (i.e., surgery or needle biopsy, spinal level of tumor) and clinical outcomes including overall survival (OS), defined as time from procedure date to date of last follow-up or date of death. All survival analyses account for the left truncated nature of the data, entering patients into the risk set at the time of their genomic sequencing report date [18]. Note that, given the left truncated nature of the data, the number at risk may increase due to varying sequencing report dates.

### 2.2. Sequencing Analysis

MSK-IMPACT is a validated, multi-targeted, FDA-authorized, next-generation sequencing assay employed to detect gene mutations and other critical genetic aberrations in both rare and common cancers [13]. Over 111,000 tumors have been profiled to date. Genomic data available on the IMPACT sequencing platform includes mutation calling, copy number alterations, and gene fusions. The genomic landscape can be displayed in an OncoPrint, facilitating representation of multiple genomic alteration events by heatmaps and other analytics summarizing the distribution of events.

### 2.3. Machine Learning Model

We applied the OncoCast algorithm, previously reported for survival prediction and stratification of patients with advanced stage lung adenocarcinoma, to the mutational data obtained from spine metastases [14]. OncoCast is an ensemble learning framework for time-to-event outcome stratification and prediction relying on elastic-net penalized Cox proportional hazard models combining both the L1 and L2 penalties of LASSO and ridge methods for variable selection, respectively [14]. OncoCast uses the variable selection frequency across all models generated to estimate relative importance of gene-level alterations as covariates. This is performed through repeatedly applying cross-validation, randomly splitting the dataset between a training set (two-thirds of the data) and a testing set (the remaining third of the data) 100 times to generate an ensemble of classifiers. The training set is used to build the model, while the testing set is used to assess the performance of the model generated and assign a predicted risk score to each patient in that set. Once all iterations have been completed, each patient in the cohort is assigned a final predicted risk as the average of the predicted risk scores received across all runs. The final predicted risk score is subsequently scaled from 0 to 10. The predicted risk score is used as a surrogate for the aggregated effect of genomic features on the outcome. This final risk score can be further stratified into clinically relevant groups using k-means clustering. The clustering process is repeated a few times, trying different numbers of centers and selecting the one that minimizes the Akaike Information Criteria. To summarize these results, for each of the primary sites we report a Kaplan–Meier plot of the risk groups that have been created along with a survival estimate summary table and a volcano plot of feature selection frequency and regression coefficients for individual genes. For the volcano plot, a positive mean coefficient is associated with decreased survival, and a negative coefficient is associated with increased survival.

### 2.4. Statistical Analysis

For each of the primary cancer sites of interest—breast, lung, prostate, and other—we included as predictors mutations, fusions and copy-number alterations from the MSK-IMPACT sequencing platform in the OncoCast ensemble learner. We excluded silent mutations from the analysis based on OncoKB annotations in cBioPortal [19,20]. We restricted the gene list to the targeted panel used in IMPACT341, the earliest version of MSK-IMPACT, including analysis of 341 genes. These genes have been profiled for all patients. All covariates are coded as binary variables, with mutations, fusions, deletions and amplifications considered as separate events for each gene. The final model also includes the OncoCast predicted risk score, age, surgery or biopsy, and spinal level. *p*-values are calculated using Fisher’s Exact Tests.

## 3. Results

Of 535 tumor samples screened, 290 were found to have high tumor purity, resulting in reliable genomic testing and five of them were excluded as they were primary tumors. Mutational profiling from bone lesions can be compromised by DNA quality and tumor content. A total of 282 samples were included in the final analysis. These included 84 breast cancer metastases, 56 non-small cell lung cancer (NSCLC), 49 prostate, and 93 from various ‘other’ primary sites. Median age of the breast, lung, prostate, and other groups were 57, 67, 70, and 60 years old respectively. The majority of tissue was obtained via minimally invasive biopsy for breast (83%), lung (54%) and prostate (86%). The thoracic spine was the most common site of disease across all groups: 49% breast, 55% lung, 41% prostate, and 41% other (Table 1).

### 3.1. Overview of the Genomic Landscape for Spinal Metastases

**Figure 1:**
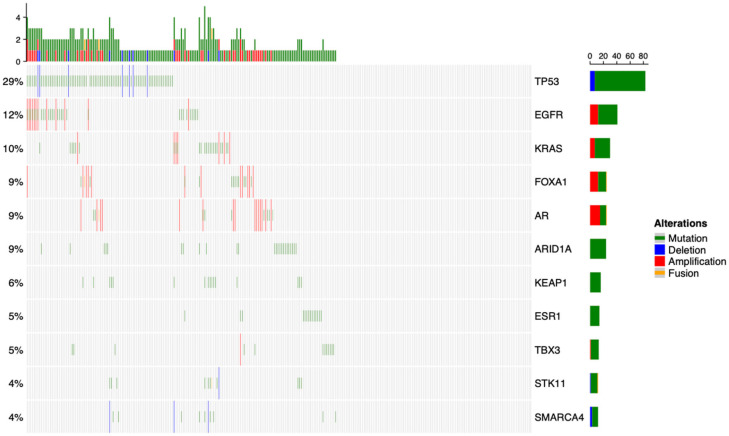
Genomic landscape of the entire cohort of 282 patients. Displayed genes are selected from a set known to be drivers of the primary tumors of interest.

Across the entire dataset, the most commonly affected gene was *TP53*, with mutations and deletions occurring in 29% of samples. *EGFR* mutations and amplifications were noted in 12% of tumors and *KRAS* mutations and amplifications in 10% (Figure 1). In analysis of the entire cohort, *KEAP1* mutation (hazard ratio 3.95, false discovery rate < 0.001) and *TP53* mutation (HR 1.80, FDR = 0.015) were associated with decreased survival (Table 2).

**Figure 2:**
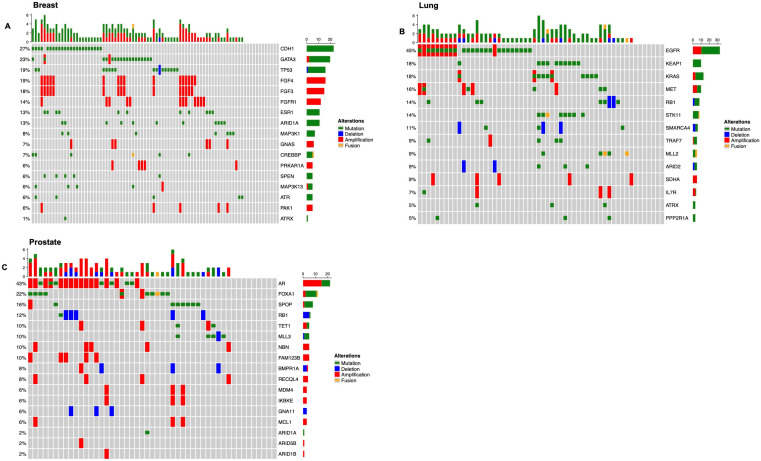
Genomic landscape of gene alterations in breast cancer: (**A**), non-small cell lung cancer (NSCLC) (**B**) and prostate cancer (**C**).

We next focused our analysis on the most common primary tumors that metastasize to the spine: breast, lung, and prostate (Figure 2). Among patients with primary breast cancer (N = 84), the median age was 57 (interquartile range (IQR) 50–66). In univariate analysis, age, tumor location, or need for surgery versus biopsy did not correlate with overall survival. Common genetic alterations are shown in Figure 2A. Presence of a *TP53* mutation was significantly associated with poor survival (HR = 3.79, FDR = 0.02). No other gene showed significance in univariate analysis. We looked at the genomic information from breast cancer primary tumors from a cohort with and without spinal metastases. The cohort that developed spinal metastases had significantly higher rates of *CDH1* mutation and significant lower rates of *TP53*, when compared to the cohort that did not develop spinal metastases (Table 3).

Among the patients with metastatic lung adenocarcinoma (N = 56), the median age was 67 years (IQR 58–72). In univariate analysis, age, tumor location, or need for surgery versus biopsy was not correlated with overall survival, although age trended toward significance (*p* = 0.06). The most common genetic alterations are shown in Figure 2B. In univariate analysis, there were six genetic alterations associated with decreased survival. These were: *IL7R* amplification (HR 15.9, FDR 0.01), *MET* amplification (HR 6.1, FDR 0.01), *RB1* mutation (HR 4.4, FDR 0.02), *TRAF7* mutation (HR 5.8, FDR 0.02), *MLL2* mutation (HR 6.0, FDR 0.04), and *SMARCA4* deletion (HR 6.1, FDR 0.04). The cohort that developed spinal metastases had significantly higher rates of *CDKN2A*, *CDKN2AP14ARF*, *CDKN2AP16INK4A* and *CDKN2B* deletion, *EGFR* and *TERT* amplification, and *EGFR* mutation when compared to the cohort that did not develop spinal metastases (Table 3).

Among the patients with metastatic prostate cancer (N = 49), the median age was 70 years (IQR 63–74). The majority of these patients (86%) had samples obtained via biopsy. In univariate analysis, age, tumor location, or surgery versus biopsy was not correlated with overall survival. The most common mutations are shown in Figure 2C. No genetic mutation was associated with survival in univariate analysis after multiple comparison adjustments. The cohort that developed spinal metastases had significantly higher rates of *AR* amplification when compared to the cohort that did not develop spinal metastases (Table 3).

### 3.2. Defining Risk Subgroups 

Next, we analyzed each primary cancer type using OncoCast to calculate a predicted risk score for survival and derive subgroups based on clinical and genomic data. Kaplan–Meier survival curves are presented for the OncoCast-defined risk subgroups for each cancer type. We additionally sought to determine the gene alterations that had prognostic significance beyond what could be identified in the univariate analysis. To determine which genes were significant contributors to overall survival, volcano plots were generated, plotting the selection frequency (importance) of each gene versus the average regression coefficient for each. A positive coefficient represents an unfavorable association with survival, while a negative coefficient represents a favorable or protective effect on overall survival. An arbitrary cutoff of 80% selection frequency was used to define prognostically relevant mutations.

#### 3.2.1. Breast Cancer

**Figure 3:**
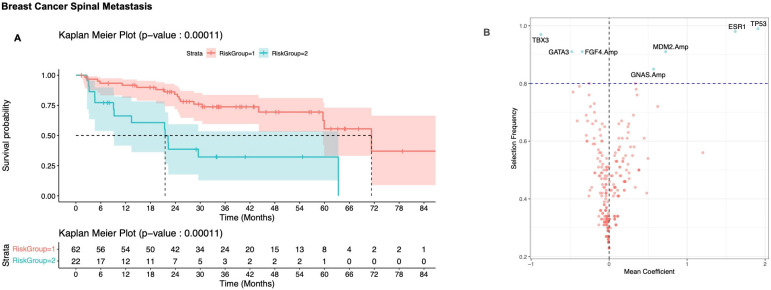
(**A**) Kaplan–Meier plot of survival for the risk stratified subgroup of breast cancer distinguished by OncoCast; 95% confidence intervals are shaded for each plot. Risk group 1 (red) represents low risk and risk group 2 (blue) represents high risk. (**B**) Volcano plot of selection frequency and regression coefficients for individual genes. Positive mean coefficient is associated with decreased survival, negative coefficient is associated with increased survival. Dashed horizontal line represents an arbitrary cut-off of 80% to determine prognostically significant genes (blue dots represent genes that are prognostically significant, while red dots represent genes that are prognostically insignificant).

OncoCast analysis was used to define two risk groups. The low-risk group had a median OS of 71 months (95% CI: 44, NR), and the high-risk group had a median OS of 22 months (95% CI: 9, NR, HR 3.3; *p* = 0.00063) (Figure 3A). *ESR1* and *TP53* mutations were highly prognostically relevant, being selected in over 90% of the models (Figure 3B). Additional prognostically relevant genes of interest included *MDM2*, *AURKA*, and *GNAS* amplification and *PIK3CA* mutation. Mutations in *TBX3* and *ATR*, as well as *FGF4* amplification, were highly selected and were associated with a survival benefit (Figure 3B). The concordance probability estimate across the 100 iterations of the breast cancer model had a median of 0.63 (interquartile range [IQR]: 0.62, 0.65).

#### 3.2.2. Non-Small Cell Lung Carcinoma

**Figure 4:**
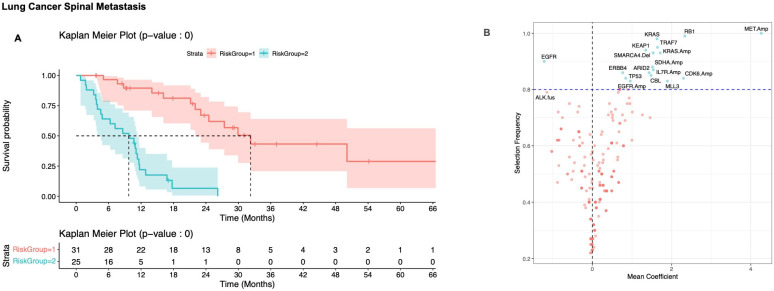
(**A**) Kaplan–Meier plot of survival for the risk stratified subgroup of NSCLC cancer distinguished by OncoCast; 95% confidence intervals are shaded for each plot. Risk group 1 (red) represents low risk and risk group 2 (blue) represents high risk. (**B**) Volcano plot of selection frequency and regression coefficients for individual genes. Positive mean coefficient is associated with decreased survival, negative coefficient is associated with increased survival. Dashed horizontal line represents an arbitrary cut-off of 80% to determine prognostically significant genes (blue dots represent genes that are prognostically significant, while red dots represent genes that are prognostically insignificant).

OncoCast analysis was used to define two risk group. The low-risk group had a median OS of 30 months (95% CI: 22, NR), and the high-risk group had a median OS of 6 months (95% CI: 0.8,11, HR 8.3; *p* < 0.001) (Figure 4A). *MET* amplification was highly prognostically relevant, being selected in all of the models. *EGFR* mutation was associated with better prognosis in OncoCast analysis, and selected in 92% of models. *ALK* fusion was also favorable and selected in 83% (Figure 4B). Overall analysis of NSCLC was characterized by a number of prognostically relevant genes that were highly represented in the models and associated with poor prognosis, including mutations and amplifications in *KRAS*, mutations or deletions in *RB1*, *TRAF7*, *KEAP1*, *ARID2*, *CBL*, *MLL3*, *TP53*, *ERBB4*, and *SMARCA4*, and amplifications of *IL7R*, *EGFR*, *BCL6*, *MAR3K13*, *TP63*, *SDHA*, and *CDK6* (Figure 4B). The concordance probability estimate across the 100 iterations of the NSCLC model had a median of 0.73 (interquartile range [IQR]: 0.70, 0.77).

#### 3.2.3. Prostate Cancer

**Figure 5:**
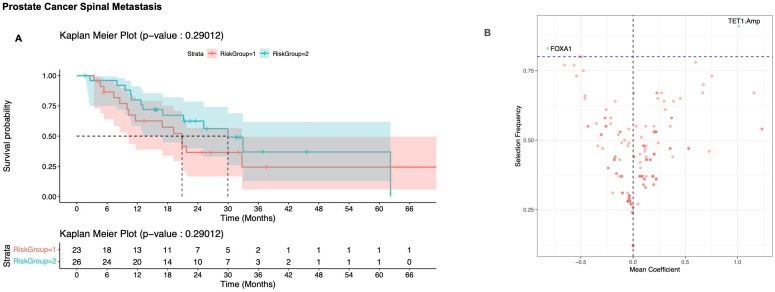
(**A**) Kaplan–Meier plot of survival for the risk stratified subgroup of prostate cancer distinguished by OncoCast; 95% confidence intervals are shaded for each plot. Risk group 1 (red) represents low risk and risk group 2 (blue) represents high risk. (**B**) Volcano plot of selection frequency and regression coefficients for individual genes. Positive mean coefficient is associated with decreased survival, negative coefficient is associated with increased survival. Dashed horizontal line represents an arbitrary cut-off of 80% to determine prognostically significant genes (blue dots represent genes that are prognostically significant, while red dots represent genes that are prognostically insignificant).

Using genomic data from prostate cancer spine tumor samples, OncoCast failed to generate meaningfully different prognostic groups (Figure 5A). *TET1* amplifications were associated with poor prognosis and *FOXA1* mutation was associated with survival benefit (Figure 5B). The concordance probability estimate across the 100 iterations of the prostate cancer model had a median of 0.62 (interquartile range [IQR]: 0.59, 0.66).

## 4. Discussion

### 4.1. Key Findings and Significance

With advances in cancer care including targeted therapies, spinal metastases may no longer be predictive of imminent mortality. Prognostication is key for tailored treatment of spinal metastases, yet there is little information on the role of genetic data in prognostication. To the best of our knowledge, this represents the largest and most comprehensive genomic analysis of metastatic spine tumors to date. We report findings on the most prevalent genetic alterations in a cohort of mixed histologies, as well as histology-specific analyses for the most common malignancies that present with spinal metastases. Utilizing the machine learning algorithm, OncoCast, we illustrate the potential of mutational profiling in informing individual prognoses from time of spine metastases sampling. The incorporation of mutation data into the tailored decision-making algorithm for those with spinal metastases will help improve accuracy of prognostication and patient prognoses, and aid in selection of multidisciplinary approaches to the treatment.

### 4.2. Clinical Implications

The treatment of spinal metastatic disease often requires a multidisciplinary approach, with treatment options ranging from systemic chemotherapy to radiation to minimally invasive interventions and finally open surgery. Key to these decisions is an understanding of the overall prognosis of these patients. High-throughput genetic sequencing is becoming widely available, and could represent a prognostic tool to assist in decision making. We have previously reported a high concordance rate between mutational profiling of primary tumors and their matching spine metastasis, particularly for driver mutations [21]. Several scoring systems based on clinical parameters have been developed and are widely utilized to aid with prognostic survival estimation for the purpose of informed decision making for the management of spine metastases. These include the Tomita Score [22], Tokuhashi Score [23], Bauer Score [24], and more recently developed tools such as the SORG Nomogram [25]. However, none of these tools incorporate tumor genetic information. The data described in this analysis from a large cohort of primary and metastatic tumors, combined with a machine learning algorithm, provides valuable insight into risk stratification of spinal metastatic disease particularly from tumors of breast, lung, and prostate origin. This open-source algorithm can be used to define patient specific prognostication based on the spine tumor genomic landscape (https://github.com/AxelitoMartin/OncoCast#oncocast-r-package (accessed on 4 October 2023). To our knowledge this is the first resource to allow incorporation of genomic data for prognostication in patients with spinal metastases. This tool can be incorporated in decision making algorithms, such as the neurologic, oncologic, mechanical, and systemic (NOMS) decision framework and other prediction models, to help improve the accuracy of prognostic scoring [3,26,27]. We believe this is a valuable tool for the cancer community. In addition to practical clinical decision making, the dataset and methodology presented here could be extended to reveal insights into molecular determinants of metastatic disease to the spine.

### 4.3. Histology-Specific Observations

Historically, breast cancer has a wealth of mutational data to guide treatment and prognosis, with stratification by *BRCA* status routinely used in clinical practice [28]. A leading prognostically relevant mutation found in our analysis was *ESR1*, which encodes the estrogen receptor. Mutations in *ESR1* have previously been established as a mechanism of resistance to aromatase inhibitor therapy [29,30]. As hormone receptor status is a critical driver of treatment in breast cancer, mutations in *ESR1* that lead to hormone resistance play a substantial role in determining treatment algorithm and overall outcome [29,31]. Our results are consistent with other reports that found these mutations to be frequent in metastatic tumors, as the ESR1 mutation is often an acquired mutation during or after the treatment with aromatase inhibitors, and associated with a worse prognosis [32,33]. Of note, the rate of *ESR1* mutation was not significantly different between the patients who developed metastatic disease and those who did not. Our data also supports a negative prognostic role for *TP53* mutation in breast cancer spinal metastases. Previous work has found an association between high TP53 staining and decreased survival in visceral breast metastases [34], while analysis of *TP53* mutational status to predict survival has produced contradictory results that may be in part due to differential response to chemotherapy regimens across *TP53* genotypes [35]. Our work demonstrates that, for spinal metastatic disease, *TP53* mutation is highly prognostically relevant and mutation confers a worse prognosis in breast cancer metastatic to the spine. However, there was a significantly decreased rate of *TP53* mutation in the cohort of patients who developed spinal metastases compared to those who did not. This could be due to early death before metastases could occur in those with *TP53* mutation.

The recent introduction of targeted therapies has revolutionized the treatment of lung adenocarcinoma [36]. Indeed, in our model, presence of druggable mutations in *EGFR* as well as *ALK* fusions appears to be protective, which is consistent with limited previously published spine metastasis data [37], and consistent with the availability of highly active targeted therapies for these mutations. Patients who developed spinal metastases were significantly more likely to have an *EGFR* mutation in the primary tumor compared to those who did not develop spinal metastases, which could be explained by increased survival in the *EGFR* mutated tumors due to targeted therapies. In a series of lung adenocarcinoma patients metastatic to the spine with mutational data for selected markers *(ALK*, *MET*, *ROS1*, *EGFR*, and *KRAS)*, the presence of clinically targetable mutations was found to be associated with increased survival [37]. In our series, amplification of the *MET* gene was found to be negatively prognostically significant in metastatic NSCLC to the spine. *MET* encodes a tyrosine kinase receptor, previously found to be upregulated as a resistance mechanism against *EGFR* inhibitors [38]. *MET* amplification is a poor prognostic indicator [39], and in experimental models has been shown to induce a more aggressive tumor phenotype [40]. Ongoing and recently completed clinical trials are exploring the concomitant use of *EGFR* and *MET* inhibitors in metastatic lung cancer [41].

In addition to known high-risk genetic mutations, our dataset also suggests previously undescribed roles for genetic mutations in spine metastatic tumors. For example, *TRAF7* mutation was found in both univariate analysis and in the OncoCast model to be prognostically significant in lung cancer metastatic to the spine. *TRAF7* has been linked to regulation of the tumor necrosis factor alpha pathway and *TP53* pathway. Genetic alternations to this gene have been previously described in meningiomas and mesotheliomas [42], but never in lung cancer or spinal or osseous metastatic disease, generating future directions for research. In the pooled dataset of 282 spine metastatic tumors, *KEAP1* was mutated in 6% of samples and was predictive of poor survival. *KEAP1* is a key component of the *Nrf2-Keap1* signaling pathway, which in both normal and cancer cells serves to protect from environmental toxins, including chemotherapy [43]. Pharmacologic targeting of the *Nrf2-Keap1* pathway is currently under investigation for a variety of diseases, including multiple sclerosis and cancer [44].

OncoCast has previously been applied in a series of 1054 patients with metastatic lung adenocarcinoma [14]. This analysis identified co-mutation of *STK11* and *KEAP1* as strong predictors of unfavorable prognosis. Interestingly, in this series of NSCLC spine metastases, the OncoCast score was driven by a distinct genomic signature, primarily by *MET* amplification (selected in 100% of models) and *RB1* mutation (selected in 99%); consistent with the prior analyses, *STK11* was selected in 75% of models and *KEAP1* in 94%. In the previously reported series of recurrent lung adenocarcinoma, the median OS in the low-risk group was 32.8 versus 7.3 months in the high-risk group [14]; this is comparable to our spine series, with a median survival of 30 months in the low-risk group and 6 months in the high-risk group.

In contrast to lung and breast cancers, OncoCast analysis yielded limited prognostic information for prostate cancer. Prostate cancer differs from breast and lung cancer in multiple ways that may impact prognostication. One distinction is the exceptionally high bone tropism of prostate cancer: if all or nearly all patients with metastatic disease have bony disease, there may be fewer discriminant determinants of outcome. Another factor is the relatively indolent course of metastatic prostate cancer relative to breast and lung cancers: for this disease, unlike others, the nature of isolated spine metastases per se may not be a primary determinant of survival. A third is the relative lack of subtype-specific driver oncogenes in prostate cancer. In contrast, lung cancers exhibit prognostic variations based on the presence of *EGFR* and *KRAS*, influenced in part due to historic availability of highly active *EGFR* tyrosine kinase inhibitors; in the breast, presence or absence of estrogen receptor expression dictates therapeutic options.

### 4.4. Limitations

This study has important limitations. This was an exploratory analysis and individual predictors of outcome identified here will ultimately need to be validated in an independent cohort of patients. While MSK-IMPACT includes a large number of cancer-relevant genes with high depth of sequencing, it is a targeted sequencing platform and therefore genes not assessed are by definition uninformative. The sample size here for individual cancer types beyond breast and lung cancer is quite limited. In addition, the rate of developing spinal metastases is quite low, and the small metastatic subset might be highly selected for. The results may be confounded by treatment heterogeneity, including the primary tumors’ genomic makeup and response to targeted therapies. It is possible that the inability to build an OncoCast model in the prostate tumor group is due to an inadequate number of samples (N = 49) rather than a lack of robust genetic predictors of survival in these patients. Other explanations for inability to build an OncoCast model in the prostate cancer group include panel limitations and non-genomic drivers such as epigenetic changes. In addition, of the 535 samples screened, 45.8% were excluded due to lack of tumor purity that prevented the ability to extract detailed genomic information. 

Our patient population is derived from a quaternary care center with a dedicated spine metastases service and therefore has access to targeted therapies, radiation therapy, and surgical options which may not be available to the community at large, possibly impacting patient outcomes. In addition, while all patients in the “non-spinal metastasis” cohort did not have spinal pathology on MSK-IMPACT, we cannot be certain that all patients had imaging to confirm the absence of spinal involvement. Finally, our data is focused on targeted DNA sequencing, without incorporating gene level expression or epigenetic markers. Future directions include external validation of the OncoCast model in independent cohorts, exploration into differences in mutation rates between primary tumors and spinal metastases, and expanded analysis, including transcriptomic and epigenetic data to complement DNA sequencing.

## 5. Conclusions

The genomic mutational landscape of spinal metastases can be used to generate prognostic groups across common tumors that metastasize to the spine. These groups are defined by genetic alterations that provide prognostic value specifically in the context of spinal metastatic disease. We have identified genetic mutations with apparent prognostic significance in these tumors that can serve as the basis for future study, as well as provide a resource of genetic mutations across 282 samples from spine metastases.

## Figures and Tables

**Table 1:** Patient characteristics.

Characteristic	Breast, N = 84 ^1^	Lung, N = 56 ^1^	Prostate, N = 49 ^1^	Other, N = 93 ^1^	*p*-Value
Age	57 (50, 66)	67 (58, 72)	70 (63, 74)	60 (50, 68)	<0.001 ^2^
Surgery or Biopsy					<0.001 ^3^
Biopsy	70 (83%)	30 (54%)	42 (86%)	38 (41%)	
Surgery	14 (17%)	26 (46%)	7 (14%)	55 (59%)	
Spinal Level					0.003 ^4^
Lumbar	25 (30%)	16 (29%)	16 (33%)	31 (33%)	
Other	2 (2.4%)	5 (8.9%)	0 (0%)	15 (16%)	
Sacral	16 (19%)	4 (7.1%)	13 (27%)	9 (9.7%)	
Thoracic	41 (49%)	31 (55%)	20 (41%)	38 (41%)	

^1^ Median (IQR); n (%). ^2^ Kruskal–Wallis rank sum test. ^3^ Pearson’s Chi-squared test. ^4^ Fisher’s Exact Test for count data with simulated *p*-value (based on 2000 replicates).

**Table 2:** Overview of the genomic landscape for spinal metastases.

Characteristic	N	HR ^1^	95% CI ^1^	*p*-Value	q-Value ^2^	Mutation Frequency
*KEAP1*	282	3.95	2.24, 6.98	<0.001 *	<0.001 **	0.06
*TP53*	282	1.80	1.26, 2.56	0.001 *	0.015 **	0.27
*KRAS*	282	1.87	1.11, 3.16	0.019 *	0.14	0.08
*CDH1*	282	0.43	0.21, 0.88	0.021 *	0.14	0.09
*GATA3*	282	0.50	0.23, 1.07	0.076	0.4	0.07
*PIK3CA*	282	0.71	0.46, 1.11	0.14	0.5	0.18
*AR*.Amp	282	1.60	0.84, 3.05	0.2	0.5	0.05
*NF1*	282	1.54	0.81, 2.94	0.2	0.5	0.06
*APC*	282	0.59	0.26, 1.33	0.2	0.5	0.06
*MLL3*	282	0.67	0.36, 1.24	0.2	0.5	0.09

^1^ HR = Hazard ratio, CI = Confidence interval. ^2^ False discovery rate correction for multiple testing. * signifies significance for *p*-value at <0.05; ** signifies significance for q-value at <0.05.

**Table 3:** Comparison of cohort with and without spine metastases.

Characteristic	No Spine Met (%), N = 5658 ^1^	Spine Met (%), N = 84 ^1^	*p*-Value ^2^	q-Value ^3^
Breast				
*TP53*	2314 (41)	15 (18)	<0.001 *	<0.001 **
*CDH1*	789 (14)	23 (27)	0.001 *	0.016 **
*ARID1A*	320 (5.7)	11 (13)	0.008 *	0.067
*MYC*.Amp	640 (11)	3 (3.6)	0.022 *	0.13
*KMT2C*	456 (8.1)	12 (14)	0.045 *	0.2
*TBX3*	306 (5.4)	9 (11)	0.048 *	0.2
Lung Adenocarcinoma				
*CDKN2A*.Del	635 (9.7)	15 (27)	<0.001 *	0.008 **
*CDKN2AP14ARF*.Del	623 (9.5)	14 (25)	<0.001 *	0.009 **
*CDKN2AP16INK4A*.Del	635 (9.7)	14 (25)	<0.001 *	0.009 **
*CDKN2B*.Del	602 (9.2)	13 (23)	0.001 *	0.012 **
*EGFR*.Amp	392 (6.0)	10 (18)	0.002 *	0.012 **
*EGFR*	1624 (25)	23 (41)	0.008 *	0.043 **
*TERT*.Amp	418 (6.4)	9 (16)	0.009 *	0.043 **
Prostate				
*AR*.Amp	320 (13)	15 (31)	0.002 *	0.034 **
*APC*	202 (8.3)	9 (18)	0.032 *	0.2
*MYC*.Amp	159 (6.6)	7 (14)	0.042 *	0.2

^1^ n (%). ^2^ Fisher’s exact test. ^3^ False discovery rate correction for multiple testing. * signifies significance for *p*-value at <0.05; ** signifies significance for q-value at <0.05.

## Data Availability

OncoCast is an open-source algorithm that can be used to define patient specific prognostication based on the spine tumor genomic landscape. The algorithm is currently available and can be found at this website: https://github.com/AxelitoMartin/OncoCast#oncocast-r-package (accessed on 4 October 2023). Information for patient samples and genes included in the MSK-IMPACT panel is available from cBioPortal at the following website [15,16,17]: https://www.cbioportal.org (accessed on 4 Ocotber 2023).

## Abbreviations

The following abbreviations are used in this manuscript:

ML	Machine learning
MSK-IMPACT	Memorial Sloan Kettering-Integrated Mutation Profiling of Actionable Cancer Targets
NSCLC	Non-small cell lung cancer
OS	Overall survival
